# *catena*-Poly[[cobalt(II)-μ-aqua-di-μ-butano­ato-κ^2^ *O*:*O*′;κ^2^ *O*:*O*] 0.7-hydrate]

**DOI:** 10.1107/S1600536811019271

**Published:** 2011-05-28

**Authors:** A. I. Fischer, V. V. Gurzhiy, A. N. Belyaev

**Affiliations:** a St Petersburg State Institute of Technology, Moskovsky pr. 26, 190013 St Petersburg, Russian Federation; b St Petersburg State University, Universitetskaya nab. 7/9, 199034 St Petersburg, Russian Federation

## Abstract

In the title coordination polymer, {[Co(C_3_H_7_COO)_2_(H_2_O)]·0.7H_2_O}_*n*_, the Co^2+^ cation is coordinated by four bridging butano­ate anions and two bridging water mol­ecules in a severely distorted octa­hedral geometry. The Co^2+^ cations are linked by means of bridging ligands into polymeric chains along [010]. These chains are further connected to each other through hydrogen bonds involving partially occupied disordered water mol­ecules; thus, sheets parallel to (001) are formed. One of the positions of disordered water O atom lies on a twofold axis. Two atoms of the aliphatic chain of one of the butanoate anions are disordered over two positions each.

## Related literature

For properties and applications of cobalt carboxyl­ates, see: Eremenko *et al.* (2009 ▶); Gates (1992 ▶); Parshall & Ittel (1992 ▶); Partenheimer (1995 ▶). For related structures, see: Jiao *et al.* (2000 ▶); Fischer *et al.* (2010 ▶).
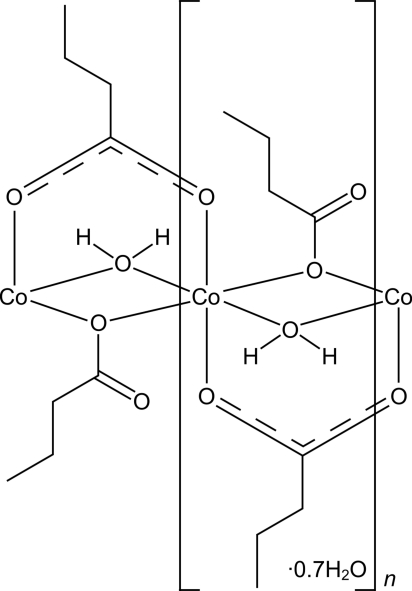


## Experimental

### 

#### Crystal data

[Co(C_4_H_7_O_2_)_2_(H_2_O)]·0.7H_2_O
*M* *_r_* = 263.75
Monoclinic, 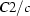

*a* = 14.8377 (13) Å
*b* = 6.2597 (7) Å
*c* = 25.743 (3) Å
β = 104.900 (3)°
*V* = 2310.6 (4) Å^3^
*Z* = 8
Mo *K*α radiation
μ = 1.49 mm^−1^
*T* = 210 K
0.10 × 0.08 × 0.03 mm

#### Data collection

Bruker APEXII CCD diffractometer
Absorption correction: multi-scan (*SADABS*; Bruker, 2007 ▶) *T*_min_ = 0.301, *T*_max_ = 0.351
12584 measured reflections
2731 independent reflections
1752 reflections with *I* > 2σ(*I*)
*R*_int_ = 0.096

#### Refinement

*R*[*F* ^2^ > 2σ(*F* ^2^)] = 0.042
*wR*(*F* ^2^) = 0.096
*S* = 0.82
2731 reflections
156 parameters
2 restraints
H atoms treated by a mixture of independent and constrained refinement
Δρ_max_ = 0.62 e Å^−3^
Δρ_min_ = −0.58 e Å^−3^

### 

Data collection: *APEX2* (Bruker, 2009 ▶); cell refinement: *SAINT* (Bruker, 2009 ▶); data reduction: *SAINT*; program(s) used to solve structure: *SHELXS97* (Sheldrick, 2008 ▶); program(s) used to refine structure: *SHELXL97* (Sheldrick, 2008 ▶); molecular graphics: *DIAMOND* (Brandenburg, 2008 ▶); software used to prepare material for publication: *publCIF* (Westrip, 2010 ▶).

## Supplementary Material

Crystal structure: contains datablocks global, I. DOI: 10.1107/S1600536811019271/ya2137sup1.cif

Structure factors: contains datablocks I. DOI: 10.1107/S1600536811019271/ya2137Isup2.hkl

Additional supplementary materials:  crystallographic information; 3D view; checkCIF report

## Figures and Tables

**Table 1:** Selected bond lengths (Å)

Co1—O3	2.027 (2)
Co1—O5	2.049 (2)
Co1—O4	2.074 (2)
Co1—O4^i^	2.105 (2)
Co1—O2	2.163 (2)
Co1—O2^ii^	2.217 (2)

Symmetry codes: (i) 
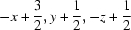
; (ii) 
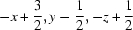
.

**Fig. 1.**
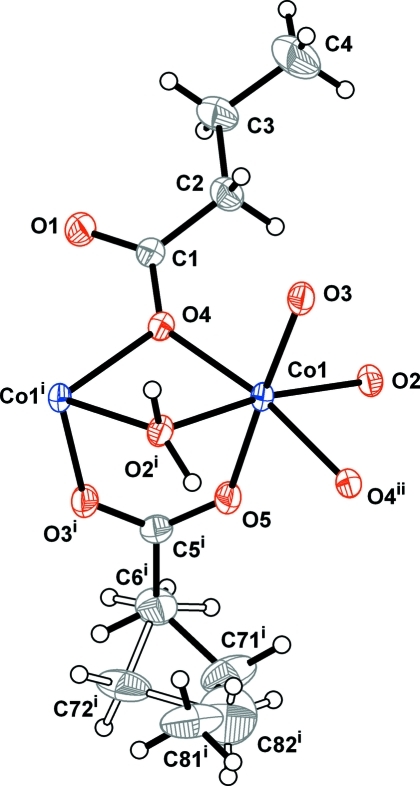
The structure of the title complex. Displacement ellipsoids of the non H-atoms are drawn at the 35% probability level. The bonds in minor component of the disordered propyl group are drawn as hollow sticks. H atoms are shown as spheres of arbitrary radius. Symmetry codes: (i) = -x + 3/2, y - 1/2, -z + 1/2; (ii) = -x + 3/2, y + 1/2, -z + 1/2.

**Fig. 2.**
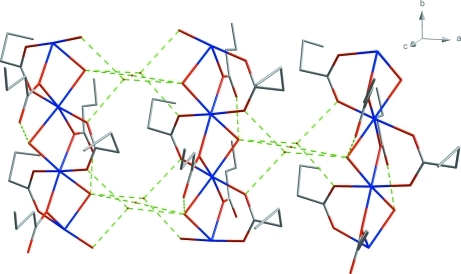
The packing diagram for the crystal of the title complex viewed down the c axis; hydrogen bonds are shown as green dashed lines. Oxygen atoms of the disordered solvate water molecules are shown as red dots. The minor components of the disordered propyl group and H atoms are omitted for clarity.

## supplementary crystallographic information

### Comment

Cobalt carboxylates are of great importance because of their application in homogeneous oxidation catalysis (Gates, 1992; Parshall & Ittel, 1992; Partenheimer, 1995), and their interesting magnetic properties (Eremenko *et al.*, 2009). Recently, we have reported on the crystal structure of the polymeric cobalt(II) propionate dihydrate, which was prepared by the reaction of cobalt(II) carbonate hydrate with aqueous propionic acid (Fischer *et al.*, 2010). We found that the use of butyric acid instead of propionic acid leads to an analogous product, {[Co(C_3_H_7_COO)_2_(H_2_O)].0.7H_2_O}*_n_* (I). This salt, named as cobalt(II) butyrate 1.7-hydrate, is interesting for us as a starting reagent for the preparation of the mixed-valence cobalt carboxylates.

The crystal structure of the title compound contains one independent Co^2+^ cation coordinated by four O atoms of four bridging butyrates and two O atoms of bridging water molecules in a severely distorted octahedral coordination (Fig. 1). The *cis-*angles about the Co atom range from 80.77 (9) to 106.47 (9)°, the Co—O bond lengths are in the range of 2.027 (2) – 2.217 (2) Å; this data correlates with the angles and the distances in cobalt(II) acetate dihydrate with similar structure (Jiao *et al.*, 2000) as well as isostructural cobalt(II) propionate dihydrate (Fischer *et al.*, 2010). The structure of the title compound features infinite chains with composition _∞_[Co(C_3_H_7_COO)_4/2_(H_2_O)_2/2_] running along [010]. The Co···Co minimum distance in the chain is equal to 3.1436 (7) Å. The bridging butyrate groups adopt two coordination modes, monodentate and *syn-syn* bidentate. The bidentate group has C—O bonds of practically equal length, 1.253 (4) and 1.254 (4) Å, whereas monodentate group has different C—O bond lengths, 1.231 (4) and 1.293 (4) Å. The chains are connected to each other through O—H···O hydrogen bonds involving partially occupied disordered water molecules; as a result, the sheets parallel to the (001) plane are produced (Fig. 2). The O61 atom of disordered solvate water molecule occupies a special position on the twofold axis.

### Experimental

To a solution of butyric acid (8.8 g, 100 mmol) in water (50 ml), an excess of fresh cobalt(II) carbonate hydrate, CoCO_3_.*x*H_2_O (*x*=0.35–1.00), (8.0 g, approximately 60 mmol) was added. The reaction mixture was periodically stirred in ultrasonic bath at room temperature until the liberation of carbon dioxide ceased. The unreacted CoCO_3_.*x*H_2_O was removed by filtration, and the filtrate was allowed to stand at room temperature for slow evaporation. After a few days, red single crystals of the title compound suitable for X-ray diffraction study precipitated. Yield 82%.

### Refinement

The propyl group of the bidentate carboxylate ligand is disordered over two positions in a 65:35 ratio. The solvate water molecule is disordered over two noneqivalent positions (O61—O62 = 0.76 (1) Å) with the total s.o.f. equal to 0.7 (Fig. 2); positions O61 and O62 were refined isotropically. The H atoms bound to O2 where located in the difference map and refined with O—H distances restrained to 0.95 (1) Å and *U*_iso_(H) set to 1.5*U*_eq_(O); other oxygen-bound H atoms where not included in refinement. Carbon-bound H-atoms were placed in calculated positions and were included in the refinement in the riding model approximation, with *U*_iso_(H) set to 1.5*U*_eq_(C) and C—H 0.96Å for the methyl groups and 1.2*U*_eq_(C) and C—H 0.97 Å for the methylene groups.

### Crystal data

**Table:**

[Co(C_4_H_7_O_2_)_2_(H_2_O)]·0.7H_2_O	*F*(000) = 1104
*M**_r_* = 263.75	*D*_x_ = 1.516 Mg m^−^^3^
Monoclinic, *C*2/*c*	Mo *K*α radiation, λ = 0.71073 Å
Hall symbol: -C 2yc	Cell parameters from 1731 reflections
*a* = 14.8377 (13) Å	θ = 2.7–23.0°
*b* = 6.2597 (7) Å	µ = 1.49 mm^−^^1^
*c* = 25.743 (3) Å	*T* = 210 K
β = 104.900 (3)°	Plate, red
*V* = 2310.6 (4) Å^3^	0.10 × 0.08 × 0.03 mm
*Z* = 8	

### Data collection

**Table:**

Bruker APEXII CCD diffractometer	2731 independent reflections
Radiation source: fine-focus sealed tube	1752 reflections with *I* > 2σ(*I*)
graphite	*R*_int_ = 0.096
φ and ω scans	θ_max_ = 27.7°, θ_min_ = 1.6°
Absorption correction: multi-scan (*SADABS*; Bruker, 2007)	*h* = −19→19
*T*_min_ = 0.301, *T*_max_ = 0.351	*k* = −8→8
12584 measured reflections	*l* = −33→33

### Refinement

**Table:**

Refinement on *F*^2^	Primary atom site location: structure-invariant direct methods
Least-squares matrix: full	Secondary atom site location: difference Fourier map
*R*[*F*^2^ > 2σ(*F*^2^)] = 0.042	Hydrogen site location: inferred from neighbouring sites
*wR*(*F*^2^) = 0.096	H atoms treated by a mixture of independent and constrained refinement
*S* = 0.82	*w* = 1/[σ^2^(*F*_o_^2^) + (0.0493*P*)^2^] where *P* = (*F*_o_^2^ + 2*F*_c_^2^)/3
2731 reflections	(Δ/σ)_max_ < 0.001
156 parameters	Δρ_max_ = 0.62 e Å^−^^3^
2 restraints	Δρ_min_ = −0.58 e Å^−^^3^

### Special details

**Table:**

Geometry. All e.s.d.'s (except the e.s.d. in the dihedral angle between two l.s. planes) are estimated using the full covariance matrix. The cell e.s.d.'s are taken into account individually in the estimation of e.s.d.'s in distances, angles and torsion angles; correlations between e.s.d.'s in cell parameters are only used when they are defined by crystal symmetry. An approximate (isotropic) treatment of cell e.s.d.'s is used for estimating e.s.d.'s involving l.s. planes.
Refinement. Refinement of *F*^2^ against ALL reflections. The weighted *R*-factor *wR* and goodness of fit *S* are based on *F*^2^, conventional *R*-factors *R* are based on *F*, with *F* set to zero for negative *F*^2^. The threshold expression of *F*^2^ > σ(*F*^2^) is used only for calculating *R*-factors(gt) *etc*. and is not relevant to the choice of reflections for refinement. *R*-factors based on *F*^2^ are statistically about twice as large as those based on *F*, and *R*- factors based on ALL data will be even larger.

### Fractional atomic coordinates and isotropic or equivalent isotropic displacement parameters (Å^2^)

**Table:**

	*x*	*y*	*z*	*U*_iso_*/*U*_eq_	Occ. (<1)
Co1	0.75276 (3)	0.06070 (7)	0.255905 (16)	0.02732 (14)	
O1	0.75332 (18)	−0.4281 (4)	0.37058 (9)	0.0446 (6)	
O2	0.69231 (15)	0.3197 (3)	0.29117 (9)	0.0306 (5)	
HO2A	0.6277 (13)	0.294 (6)	0.2786 (13)	0.046*	
HO2B	0.715 (2)	0.410 (5)	0.3222 (10)	0.046*	
O3	0.88459 (16)	0.1401 (3)	0.29628 (9)	0.0383 (6)	
O4	0.75018 (16)	−0.2058 (3)	0.30335 (8)	0.0330 (5)	
O5	0.61852 (16)	−0.0070 (3)	0.21419 (9)	0.0382 (6)	
O61	0.5000	0.3285 (15)	0.2500	0.073 (3)*	0.60
O62	0.4987 (6)	0.3237 (17)	0.2794 (4)	0.083 (2)*	0.40
C1	0.7659 (2)	−0.2489 (5)	0.35404 (13)	0.0318 (8)	
C2	0.8038 (3)	−0.0718 (6)	0.39244 (13)	0.0452 (9)	
H2A	0.7651	0.0535	0.3814	0.054*	
H2B	0.8659	−0.0373	0.3893	0.054*	
C3	0.8095 (4)	−0.1141 (7)	0.45058 (15)	0.0655 (13)	
H3A	0.8476	−0.2399	0.4619	0.079*	
H3B	0.7474	−0.1450	0.4543	0.079*	
C4	0.8496 (4)	0.0687 (9)	0.48730 (18)	0.0932 (18)	
H4A	0.9131	0.0923	0.4864	0.140*	
H4B	0.8474	0.0349	0.5233	0.140*	
H4C	0.8137	0.1956	0.4756	0.140*	
C5	0.9218 (2)	0.3213 (6)	0.30284 (12)	0.0318 (7)	
C6	1.0235 (2)	0.3339 (7)	0.33334 (17)	0.0537 (11)	
H6A	1.0615	0.3540	0.3082	0.064*	0.65
H6B	1.0421	0.2010	0.3524	0.064*	0.65
H6C	1.0476	0.4689	0.3241	0.064*	0.35
H6D	1.0544	0.2149	0.3210	0.064*	0.35
C71	1.0404 (5)	0.5282 (15)	0.3753 (3)	0.079 (3)	0.65
H71A	1.1063	0.5620	0.3874	0.095*	0.65
H71B	1.0070	0.6546	0.3590	0.095*	0.65
C81	1.0027 (8)	0.450 (3)	0.4231 (4)	0.092 (4)	0.65
H81A	1.0102	0.5616	0.4495	0.138*	0.65
H81B	1.0369	0.3262	0.4390	0.138*	0.65
H81C	0.9378	0.4150	0.4103	0.138*	0.65
C72	1.0412 (9)	0.272 (3)	0.3901 (5)	0.072 (4)	0.35
H72A	1.1054	0.2295	0.4055	0.087*	0.35
H72B	0.9991	0.1616	0.3960	0.087*	0.35
C82	1.014 (3)	0.536 (5)	0.4137 (14)	0.128 (16)*	0.35
H82A	0.9483	0.5481	0.4091	0.192*	0.35
H82B	1.0349	0.6452	0.3933	0.192*	0.35
H82C	1.0459	0.5517	0.4510	0.192*	0.35

### Atomic displacement parameters (Å^2^)

**Table:**

	*U*^11^	*U*^22^	*U*^33^	*U*^12^	*U*^13^	*U*^23^
Co1	0.0312 (2)	0.0181 (2)	0.0311 (2)	0.0017 (2)	0.00511 (17)	−0.00010 (19)
O1	0.0666 (17)	0.0335 (14)	0.0332 (13)	−0.0048 (14)	0.0119 (11)	0.0038 (12)
O2	0.0299 (12)	0.0232 (11)	0.0383 (13)	−0.0021 (11)	0.0079 (10)	−0.0033 (10)
O3	0.0349 (13)	0.0251 (12)	0.0491 (15)	0.0021 (10)	0.0005 (11)	−0.0009 (10)
O4	0.0445 (14)	0.0238 (11)	0.0284 (13)	0.0018 (11)	0.0054 (10)	0.0019 (9)
O5	0.0379 (13)	0.0244 (13)	0.0465 (14)	0.0047 (10)	0.0002 (11)	−0.0018 (9)
C1	0.0345 (19)	0.0289 (18)	0.0308 (19)	0.0022 (14)	0.0062 (15)	0.0002 (13)
C2	0.059 (2)	0.041 (2)	0.0350 (19)	−0.006 (2)	0.0108 (17)	−0.0055 (17)
C3	0.091 (3)	0.065 (3)	0.035 (2)	−0.005 (3)	0.006 (2)	−0.0091 (19)
C4	0.134 (5)	0.090 (4)	0.049 (3)	−0.013 (4)	0.013 (3)	−0.028 (3)
C5	0.0287 (17)	0.0374 (19)	0.0292 (18)	−0.0027 (16)	0.0070 (14)	−0.0018 (14)
C6	0.030 (2)	0.064 (3)	0.062 (3)	0.001 (2)	0.0039 (18)	0.002 (2)
C71	0.040 (4)	0.106 (7)	0.080 (6)	−0.007 (4)	−0.004 (4)	−0.036 (5)
C81	0.072 (6)	0.142 (14)	0.053 (5)	0.003 (8)	−0.004 (5)	−0.029 (8)
C72	0.040 (7)	0.113 (14)	0.051 (8)	−0.006 (7)	−0.010 (6)	0.015 (8)

### Geometric parameters (Å, °)

**Table:**

Co1—O3	2.027 (2)	C4—H4A	0.9599
Co1—O5	2.049 (2)	C4—H4B	0.9599
Co1—O4	2.074 (2)	C4—H4C	0.9599
Co1—O4^i^	2.105 (2)	C5—O5^i^	1.253 (4)
Co1—O2	2.163 (2)	C5—C6	1.512 (5)
Co1—O2^ii^	2.217 (2)	C6—C72	1.468 (13)
O1—C1	1.231 (4)	C6—C71	1.604 (8)
O2—Co1^i^	2.217 (2)	C6—H6A	0.9700
O2—HO2A	0.943 (18)	C6—H6B	0.9700
O2—HO2B	0.966 (18)	C6—H6C	0.9702
O3—C5	1.254 (4)	C6—H6D	0.9699
O4—C1	1.293 (4)	C71—C81	1.555 (17)
O4—Co1^ii^	2.105 (2)	C71—H71A	0.9700
O5—C5^ii^	1.253 (4)	C71—H71B	0.9700
O61—O62^iii^	0.763 (10)	C81—H81A	0.9599
O61—O62	0.763 (10)	C81—H81B	0.9599
O62—O62^iii^	1.53 (2)	C81—H81C	0.9599
C1—C2	1.496 (4)	C72—C82	1.84 (4)
C2—C3	1.501 (5)	C72—H72A	0.9700
C2—H2A	0.9700	C72—H72B	0.9700
C2—H2B	0.9700	C82—H82A	0.9599
C3—C4	1.506 (6)	C82—H82B	0.9599
C3—H3A	0.9700	C82—H82C	0.9599
C3—H3B	0.9700		
			
O3—Co1—O5	177.71 (9)	H4B—C4—H4C	109.5
O3—Co1—O4	93.25 (9)	O5^i^—C5—O3	125.0 (3)
O5—Co1—O4	88.83 (9)	O5^i^—C5—C6	117.4 (3)
O3—Co1—O4^i^	92.23 (9)	O3—C5—C6	117.6 (3)
O5—Co1—O4^i^	85.85 (9)	C72—C6—C5	113.7 (6)
O4—Co1—O4^i^	170.17 (8)	C72—C6—C71	64.7 (7)
O3—Co1—O2	92.90 (9)	C5—C6—C71	110.6 (4)
O5—Co1—O2	85.58 (9)	C72—C6—H6A	135.4
O4—Co1—O2	106.47 (9)	C5—C6—H6A	109.5
O4^i^—Co1—O2	81.38 (9)	C71—C6—H6A	109.5
O3—Co1—O2^ii^	90.30 (9)	C72—C6—H6B	46.7
O5—Co1—O2^ii^	90.99 (9)	C5—C6—H6B	109.5
O4—Co1—O2^ii^	80.77 (9)	C71—C6—H6B	109.5
O4^i^—Co1—O2^ii^	91.05 (8)	H6A—C6—H6B	108.1
O2—Co1—O2^ii^	171.88 (6)	C72—C6—H6C	119.3
Co1—O2—Co1^i^	91.73 (8)	C5—C6—H6C	107.5
Co1—O2—HO2A	103 (2)	C71—C6—H6C	60.0
Co1^i^—O2—HO2A	114 (2)	H6A—C6—H6C	53.8
Co1—O2—HO2B	134 (2)	H6B—C6—H6C	142.7
Co1^i^—O2—HO2B	87 (2)	C72—C6—H6D	98.2
HO2A—O2—HO2B	119 (3)	C5—C6—H6D	106.4
C5—O3—Co1	128.7 (2)	C71—C6—H6D	142.9
C1—O4—Co1	137.4 (2)	H6A—C6—H6D	58.5
C1—O4—Co1^ii^	123.4 (2)	H6B—C6—H6D	53.8
Co1—O4—Co1^ii^	97.58 (9)	H6C—C6—H6D	110.7
C5^ii^—O5—Co1	132.3 (2)	C81—C71—C6	105.6 (8)
O62^iii^—O61—O62	175 (3)	C81—C71—H6C	142.4
O61—O62—O62^iii^	2.3 (13)	C6—C71—H6C	36.9
O1—C1—O4	122.3 (3)	C81—C71—H71A	110.6
O1—C1—C2	120.8 (3)	C6—C71—H71A	110.6
O4—C1—C2	116.9 (3)	H6C—C71—H71A	92.6
C1—C2—C3	116.3 (3)	C81—C71—H71B	110.6
C1—C2—H2A	108.2	C6—C71—H71B	110.6
C3—C2—H2A	108.2	H6C—C71—H71B	87.8
C1—C2—H2B	108.2	H71A—C71—H71B	108.7
C3—C2—H2B	108.2	C6—C72—C82	95.4 (14)
H2A—C2—H2B	107.4	C6—C72—H72A	112.7
C2—C3—C4	113.8 (4)	C82—C72—H72A	112.7
C2—C3—H3A	108.8	C6—C72—H72B	112.7
C4—C3—H3A	108.8	C82—C72—H72B	112.7
C2—C3—H3B	108.8	H72A—C72—H72B	110.2
C4—C3—H3B	108.8	C72—C82—H82A	109.5
H3A—C3—H3B	107.7	C72—C82—H82B	109.5
C3—C4—H4A	109.5	H82A—C82—H82B	109.5
C3—C4—H4B	109.5	C72—C82—H82C	109.5
H4A—C4—H4B	109.5	H82A—C82—H82C	109.5
C3—C4—H4C	109.5	H82B—C82—H82C	109.5
H4A—C4—H4C	109.5		

Symmetry codes: (i) −*x*+3/2, *y*+1/2, −*z*+1/2; (ii) −*x*+3/2, *y*−1/2, −*z*+1/2; (iii) −*x*+1, *y*, −*z*+1/2.
